# Phytochemical, Antimicrobial, and Antioxidant Profiles of *Duranta erecta* L. Parts

**DOI:** 10.1155/2019/8731595

**Published:** 2019-10-24

**Authors:** Shadrack Donkor, Christopher Larbie, Gustav Komlaga, Benjamin Obukowho Emikpe

**Affiliations:** ^1^Applied Radiation Biology Centre, Radiological and Medical Sciences Research Institute, Ghana Atomic Energy Commission, Legon, Accra, Ghana; ^2^Department of Biochemistry and Biotechnology, Kwame Nkrumah University of Science and Technology (KNUST), Kumasi, Ghana; ^3^Department of Pharmacognosy, Kwame Nkrumah University of Science and Technology (KNUST), Kumasi, Ghana; ^4^Department of Veterinary Pathology, University of Ibadan, Ibadan, Nigeria; ^5^Department of Pathobiology, School of Veterinary Medicine, Kwame Nkrumah University of Science and Technology (KNUST), Kumasi, Ghana

## Abstract

The use of plant-based medicine is popular amongst individuals and communities in developing countries. *Duranta erecta* has been used in Africa and Asia to treat a wide range of diseases. This study evaluated the phytochemical profile and antioxidant and antimicrobial activities of *D. erecta* to ascertain its health benefits in traditional medicine. Phytochemical constituents and antimicrobial effect of the hydroethanolic extract of *D. erecta* leaves (DRL), unripe fruits (DRU), and ripe fruits (DRR) were investigated by standard methods. Elemental analyses were carried out by atomic absorption spectroscopy (AAS) on the raw sample and extract. FTIR and UV-VIS spectroscopy were used to identify functional groups. Extracts were screened for their possible antioxidant activities by three tests. The total phenolic and total tannin contents were evaluated by using the Folin–Ciocalteu method. Total flavonoid content was determined by the aluminium chloride colorimetric assay method. The antioxidant activities were evaluated using the DPPH scavenging activity. The results of phytochemical screening showed the presence of triterpenoids, sterols, alkaloids, flavonoids, saponins, glycosides, and tannins. FTIR analysis revealed the presence of alcohols, phenols, alkanes, aldehydes, ketones, aromatics, aliphatic amines, aromatic amines, amides, carboxylic acids, esters, nitro compounds, alkynes, primary and secondary amines, and alkyl halides. Iron, zinc, and copper were also detected. Total phenolic and tannin contents ranged from 2.20 ± 0.15 to 14.54 ± 0.29 mg gallic acid equivalent (GAE)/100 g and 3.55 ± 0.07 to 13.82 ± 0.04 mg GAE/100 g, respectively. Total flavonoid content varied from 41.76 ± 0.96 to 343.49 ± 3.45 *μ*g quercetin equivalent (QE)/100 g. The highest DPPH scavenging activity was recorded in the methanolic fraction of the leaves. The antimicrobial assay of the extract or fractions recorded no activity against the test organisms. The outcome of this study affirmed that *D. erecta* contains phytochemicals and bioactive compounds that could be of health benefit.

## 1. Introduction

The use of medicinal plants has become popular amongst individuals and communities in developing countries. There has been an upsurge in the use of herbal medicines because they are affordable, accessible, culturally acceptable, and effective and supposedly have lesser side effects as compared with synthetic drugs found in Africa [1]. Plants produce various bioactive compounds which contain components of therapeutic value making them a rich source of medicines. Approximately, 25% of prescription drugs dispensed in the United States are derived from plant sources [2]. The various components confer specific distinctiveness and properties to plants.

In folk medicine, plants are important source of natural remedies used to treat many diseases. Overdependence on traditional folk medicine has brought to light the issue of sustaining our fast-degrading forest. In Ghana, most of the traditional medicine practitioners depend on our forests for their trade, while our backyards are overgrown with ornamental plants which are equally good source of herbal medicine. Thus, if we want to conserve our forest for sustainable development, then there is the need to screen ornamental plants for biological and pharmacological properties by researchers. This is part of studies identifying alternative use for ornamental plant [3].

*Duranta*belongs to the Verbenaceae family and it comprises 35 species. It is a native plant of Asia, Africa, and South and Central America [4]. *Duranta erecta* (synonymous to *D. repens*), commonly known as “golden dew drop,” is an upright scrambling shrub with a height of 1–3 m. It is grown as a hedge or ornamental plants in various homes in Ghana. Phytoconstituents including coumarinolignoids, (E)-cinnamic acid, (E)-p-methoxycinnamic acid, and lamiide have been isolated from the genus *Duranta* [5, 6]. A number of bioactive compounds such as *β*-sitosterol, naringenin, acteoside, lamiide, sucrose, and raffinose have been identified and isolated from *Duranta repens* [7]. Different parts of the plant are used to treat variety of diseases. The fruit and leaves are used in traditional folk medicine for the treatment of malaria, intestinal worms, and abscess and, in some cases, serve as vermifuge or diuretic. *D. erecta* is said to possess antitumor activity and significant antibacterial activity [8]. It has numerous natural uses including antifungal and insecticidal properties [9]. The ethyl acetate extract of leaves reportedly exhibited significant antiplasmodial activity against the chloroquine-sensitive and chloroquine-resistant strains of *Plasmodium falciparum* [5]. Methanolic extracts of different parts such as leaves, stem, and roots of *D. erecta* exhibited antifungal properties against *Aspergillus flavus*, *Alternaria* sp., *Penicillium* sp., *Rhizopus* sp., and *Trichoderma* sp., [10]. The aim of the current study was to carry out the phytochemical and antioxidant screening of *D. erecta* grown as hedges in Ghana as a justification for its use in traditional medicine.

## 2. Materials and Methods

### 2.1. Chemicals

Folin—Ciocalteu phenol reagent (FCR); gallic acid; 2, 2-diphenyl^−1^-picrylhydrazyl hydrate (DPPH); quercetin; ascorbic acid; and DMSO were from Sigma. AlCl_3_, Na_2_CO_3_, CH_3_COOK, HClO_4_, CHCl_3_, NaOH, FeCl_3,_ and NaCl were from Merck (Darmstadt, Germany). Ethanol, methanol, petroleum ether, ethyl acetate, nitric acid, and sulphuric acid were from Janssen Chimica (Beerse, Belgium). Nutrient agar, Mueller-Hinton agar, and chloramphenicol were procured from Oxoid, England. All chemicals were of analytical grade.

### 2.2. Plant Collection and Authentication

Plant materials (leaves, ripe, and unripe fruits) were collected from KNUST campus in October 2017 and authenticated by Dr. George Sam at the Department of Herbal Medicine, Faculty of Pharmaceutical Sciences, KNUST, and the specimen was deposited in the faculty's herbarium (voucher number KNUST/HM1/2017/L011).

### 2.3. Preparation and Extraction of Plant Material

The leaves and fruits of *D. erecta* were washed under running tap water to remove dust and other extraneous substances. They were subsequently dried under shade for 4 weeks. The shade-dried parts of the plant were coarsely powdered using a grinder (Waring, USA). 60 g of the pulverized samples were extracted with 50% ethanol by cold maceration for 48 hours at room temperature on a shaker (Rocking Laboratory Shaker). The mixture was separated by centrifugation, and each supernatant was evaporated under reduced pressure using a rotary evaporator (Buchi R-205, Switzerland). The extracts were dried using a vacuum freeze dryer (Heto PowerDry LL3000, UK) to obtain the respective crude extracts which included *D. erecta* leaves and unripe and ripe fruits. 15 g of each crude extract was sequentially extracted with solvents of increasing polarity starting with petroleum ether, followed by ethyl acetate and methanol. The residual portion was designated as hydro fraction. The various fractions, namely, petroleum ether, ethyl acetate, methanol, and hydro fractions were all air-dried at room temperature and used for the tests.

### 2.4. Phytochemical Screening

The powdered samples of *D. erecta* leaves, unripe and ripe fruits, and respective crude extracts were subjected to qualitative analysis for identification of tannins (Gelatin test), glycosides (Molisch's test), alkaloids (Dragendorff's reagents), coumarins (FeCl_3_ test), saponins (foam test), flavonoids (alkaline reagent), triterpenoids (Salkowski's test), and sterols (Liebermann–Burchard test) according to the standard methods described by Harborne [11] and Sazada et al. [12].

### 2.5. Heavy Metal Analysis

Prior to sample analysis, the glassware used for the analysis was soaked overnight in 10% (v/v) nitric acid, followed by washing with 10% (v/v) hydrochloric acid, then rinsed with double-distilled water and dried to eliminate interference. Accurately, 1 g of each of the extracts and the raw samples were weighed into a 50 mL digestion tube. Each sample was wet-digested using a mixture of 1 mL of H_2_O, 2 mL of HCl, 5 mL of 1 : 1 HNO_3_ : HClO_4_, and 2 mL of H_2_SO_4_ for 20 minutes. The mixture was heated in a digestion block at 150°C. The digested sample was allowed to cool and diluted with 50 mL of distilled water. The digests were analysed to determine the levels of heavy metals in each extract. Heavy metals including lead, copper, nickel, zinc, and iron were analysed using an atomic absorption spectrometer (Varian AA 240FS) with a long path air acetylene burner and cathode lamp for respective metals.

### 2.6. UV and FTIR Spectroscopic Analysis

The fractions of leaves and fruits were diluted to 1 : 10 with respective solvents. The fractions were scanned in the wavelength ranging from 200 to 700 nm using a double-beam ultraviolet spectrophotometer (PerkinElmer, USA) to detect the characteristic peaks. 10 mg of the dried extracts was encapsulated in 100 mg of KBr pellet to prepare translucent sample discs. The powdered sample of each extract was loaded in a FTIR spectroscope (UATR Spectrum 2, PerkinElmer) with a scan range from 400 to 4000 cm^−1^ and a resolution of 4 cm^−1^. The peak values of the UV and FTIR were recorded.

### 2.7. Determination of Total Phenolic Content

The total phenolic content of the fractions was assessed by Folin–Ciocalteu (FC) procedure [13] with some modifications. Gallic acid was used as the standard. Approximately, 50 *μ*L of the extract was mixed with 3 mL of distilled water and 250 *μ*L of FC reagent. The mixture was allowed to stand for 5 minutes, and then 750 *μ*L of 20% Na2CO3 was added. The resulting mixture was vigorously vortexed for 2 min and then incubated for 30 min at room temperature. The absorbance of the solution was measured at 760 nm using a UV-VIS spectrophotometer ( UV1201, Shimadzu, Japan). All determinations were performed in triplicate. Gallic acid (0.2 mg·mL^−1^, 0.4 mg·mL^−1^, 0.6 mg·mL^−1^, 0.8 mg·mL^−1^, and 1 mg·mL^−1^) was used as standard to prepare a calibration curve from which polyphenolic content in terms of the gallic acid equivalent in one gram of each extract was determined. The total phenolic content was calculated from the calibration curve and final results expressed as mg GAE/100 g DM.

### 2.8. Determination of Total Flavonoid Content

The aluminium chloride colorimetric assay method [14] was employed to evaluate total flavonoid content (TFC) using quercetin as standard. An aliquot of 500 *μ*L of the extract and fractions were mixed with 1.5 mL of 99.9% ethanol (EtOH), 100 *μ*L of 1 M potassium acetate, 100 *μ*L of 10% aluminium chloride, and 3 mL of distilled water. The mixture was shaken vigorously and left to stand in dark at room temperature. The resulting mixtures were incubated for 30 min at room temperature and corresponding absorbance measured at 415 nm. All determinations were carried out in triplicate. A standard calibration curve was constructed using quercetin standard solutions of 20 *μ*g·mL^−1^, 40 *μ*g·mL^−1^, 60 *μ*g·mL^−1^, 80 *μ*g·mL^−1^, 100 *μ*g·mL^−1^, and 120 *μ*g·mL^−1^. 500 *μ*L of each standard was treated in the same manner as the samples above to generate calibration curve. The total flavonoid content of each extract was determined from the curve and the results recalculated and expressed as *μ*g QE/100 g DM.

### 2.9. Determination of Total Tannins

The amount of tannins in plant extract and fractions was determined by the Folin–Ciocalteu method with slight modifications [13]. 100 *μ*L of the sample extract and fractions was added to 5 mL of distilled water, 500 *μ*L of Folin–Ciocalteu reagent, 1 mL and of 35% Na_2_CO_3_ solution. The mixture was shaken well and kept at room temperature for 30 min. A set of reference standard solutions of gallic acid (0.2, 0.4, 0.6, 0.8, and 1 mg·mL^−1^) were prepared in the same manner as described earlier. Absorbance for test and standard solutions were measured against the blank at 725 nm with an UV/Visible spectrophotometer. The total tannin content was determined from the calibration curve, and the results expressed tannin content was in terms of mg of GAE/100 g DM.

### 2.10. Determination of Antioxidant Activity

The free radical scavenging ability of the extracts against DPPH free radical was evaluated as described by Oliveira et al. [15], with some modifications. Briefly, 200 *μ*L of each extract was added to 3.8 mL of 0.004% DPPH methanolic solution. After 60 min of incubation at room temperature in dark, the absorbance was measured at 517 nm. A blank sample containing only methanol was used to zero the spectrophotometer. Each experiment was performed in triplicate.

Inhibition (I %) was calculated as follows:

(1) $$\begin{array}{c} I\%=\left[\frac{Abs_{0}-Abs_{1}}{Abs_{0}}\right]\;*\;100,\\ Abs_{0}=\text{absorbance of }0.004\%\text{ DPPH without analyte,}\\ Abs_{1}=\text{absorbance of }0.004\%\text{ DPPH plus the test compound}. \end{array}$$

### 2.11. Preparation of Culture Media and Inoculum

The media used in the assay comprised nutrient agar (Oxoid, CM0003, England), bacteriological peptone (Sigma-Aldrich, P0556, Germany), and Mueller-Hinton agar (Oxoid, CM0337, Oxoid Ltd, England). The media were prepared according to the manufacturer's instructions.

#### 2.11.1. Preparation of Inoculum

The assay factored nine bacteria and yeast isolates. The stock cultures of *Pseudomonas aeroginosa* (ATCC 27853), *Escherichia coli* (ATCC 25922), *Salmonella typhi* (ATCC 19430), *Staphylococcus aureus* (ATCC 25923), *Proteus mirabilis* (ATCC 49565), *Candida albicans*, *Klebsiella pneumonia* (ATCC 33495), *Paratyphi* B, and *Staphylococcus saprophyticus* (ATCC 15305) were subcultured onto fresh nutrient agar (Oxoid, CM0003, England) plates to obtain pure colonies. Single colonies of each organism were suspended in test tubes containing 5 mL of sterilized bacteriological peptone (Sigma-Aldrich, P0556, Germany) and incubated at 37°C for 10–18 hours to attain the turbidity of 0.5 McFarland standards [16]. The turbidity of the actively growing broth cultures which were not in conformity to the standard (exceeded the standard) were adjusted with sterile bacteriological peptone to obtain turbidity optically comparable with that of 0.5 McFarland standard (approximately 1-2 × 108 CFU/mL for *E. coli* ATCC 25922) [17].

#### 2.11.2. Preparation of Plant Fractions for the Bioassays

Considering the preparation of stock solutions for the bioassays, 100 mg·mL^−1^, 200 mg·mL^−1^, and 300 mg·mL^−1^ of the fractionated plant extracts were reconstituted using 20% v/v DMSO (Sigma, D5879, Germany) resulting in percentage concentrations of 10%, 20%, and 30% for the determination of antimicrobial activity. The crude extract was dissolved in sterile distilled water for the aqueous extract preparation of same concentrations. The stock solutions were stored in a refrigerator until needed.

#### 2.11.3. Antimicrobial Activity Assay (Agar Diffusion Method)

The antimicrobial activity was carried out using the agar diffusion method [17]. The inoculums were inoculated by swabbing onto sterile Mueller-Hinton agar (Oxoid, CM0337, Oxoid Ltd, England). A sterilized cock borer of an internal diameter of about 4 mm was used to punch six holes in the medium. The plant extracts were dispensed into the holes to a volume of 100 *μ*L. The positive control was an antibiotic disc of 30 *μ*g chloramphenicol (Oxoid, CT00143 Oxoid Ltd, England), and 20% v/v DMSO (Sigma, D5879, Germany) was used as negative control in a triplicate fashion. The plates were refrigerated for about 4 h to allow for absolute diffusion of the extract and incubated at 37°C for 24 h, after which the diameter of each zone of inhibition was measured in millimetres (mm) with a sterilized ruler.

### 2.12. Data Analysis

The results were expressed as mean ± standard error mean (SEM) and compared by one-way analysis of variance followed by Tukey's multiple comparison test at the 95% significant level using GraphPad Prism version 6.0.

## 3. Results and Discussion

### 3.1. Results

#### 3.1.1. Phytochemical Constituents

Qualitative phytochemical screening of raw leaves and fruits of *D. erecta* indicated presence of tannins, glycosides, saponins, flavonoids, and triterpenoids. Alkaloids were present in unripe fruits only while coumarins were not detected in any of the parts of *D. erecta* (Table 1).

#### 3.1.2. Heavy Metal Content in *D. erecta*

The concentration levels of Ni and Pb were below detectable limits of 1 × 10^−5^ mg·L^−1^ for the leaves and fruits. The highest mean concentration for Fe (2.17 ± 0.05 mg·L^−1^) was found in leaves (raw) and the lowest (0.33 ± 0.01 mg·L^−1^) in the ripe fruit extracts (Table 2). The permissible limit of Fe in medicinal plants is 20 mg·mL^−1^ [17].

#### 3.1.3. UV-Vis Spectroscopic Analysis

The UV-Vis spectroscopy of extract/fractions of *D. erecta* was done at a range of 200 to 700 nm. The number of peaks and maximum absorption range is shown in Table 3.

#### 3.1.4. FTIR Spectra

**Figure 1:**
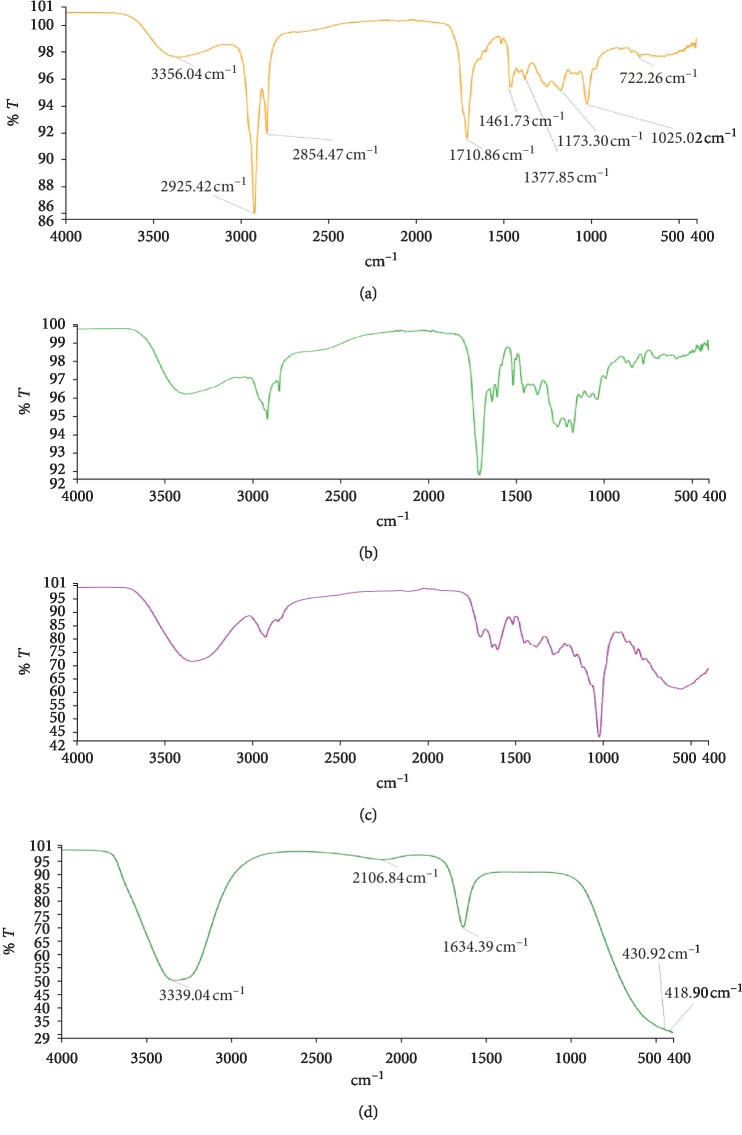
FTIR spectrum of petroleum ether (a), ethyl acetate (b), methanolic fraction (c), and hydro fraction (d) of *D. erecta* leaf extract.

**Figure 2:**
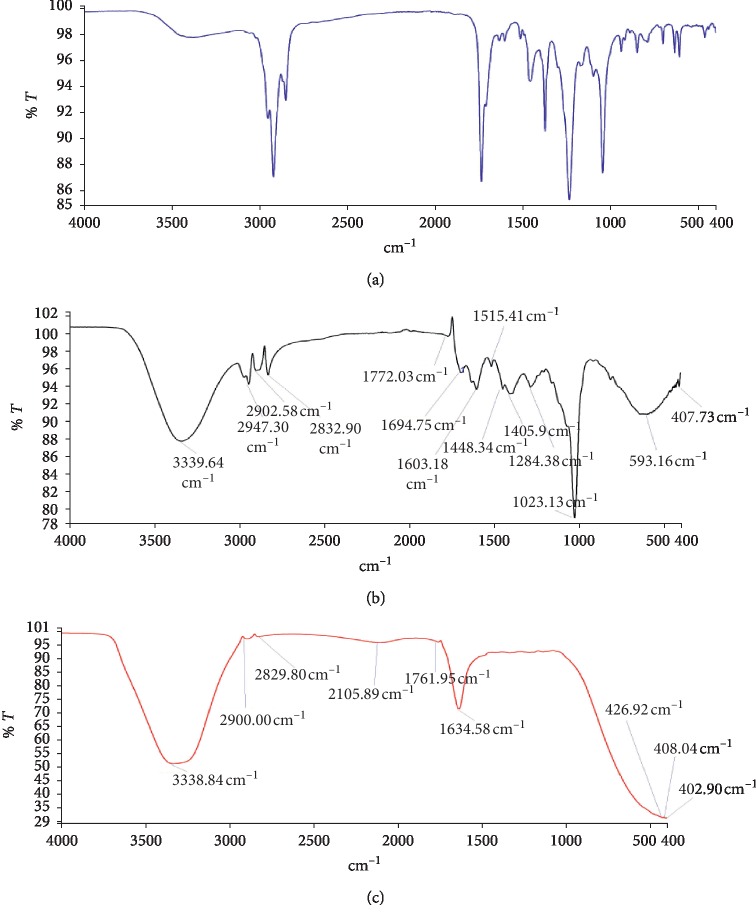
FTIR spectrum of ethyl acetate (a), methanolic fraction (b), and hydro fraction (c) of *D. erecta* unripe fruit extract.

**Figure 3:**
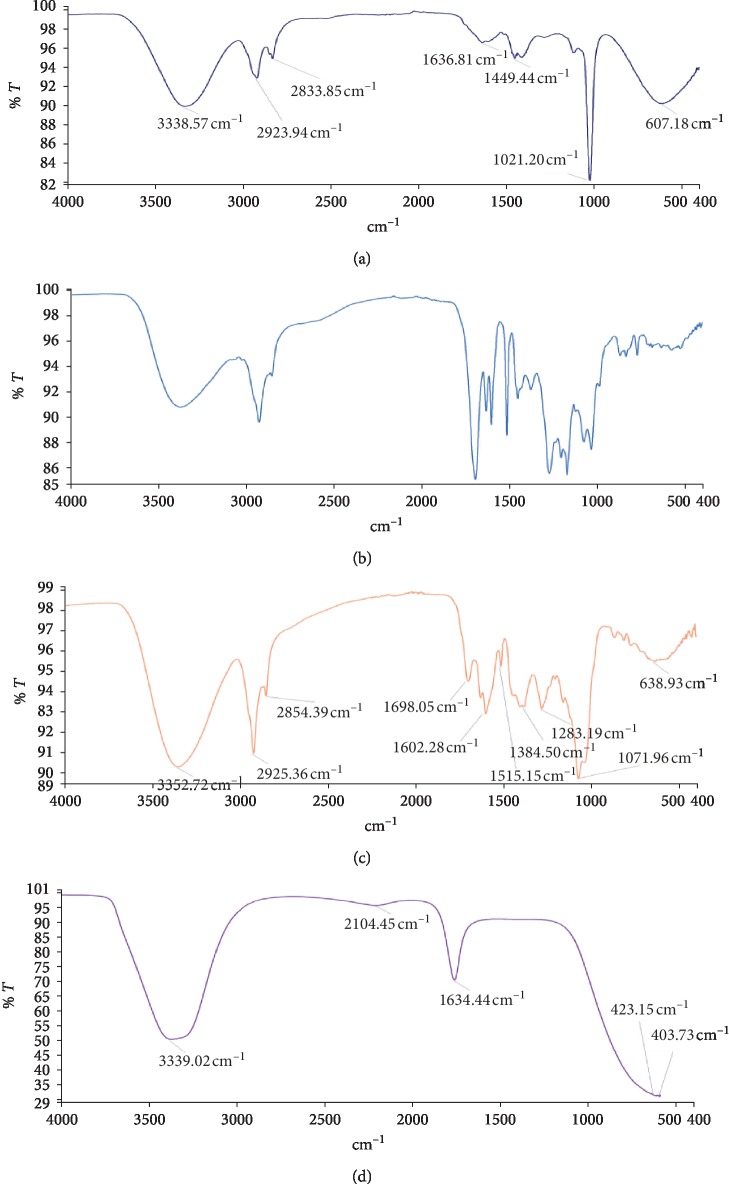
FTIR spectrum of petroleum ether (a), ethyl acetate (b), methanolic (c), and hydro fraction (d) of *D. erecta* ripe fruit extract.

The FTIR spectra are shown in Figures 1–3, and these enabled the identification of possible functional groups of the active components present in the fractions of *D. erecta*.

#### 3.1.5. Total Phenolic Content

**Figure 4:**
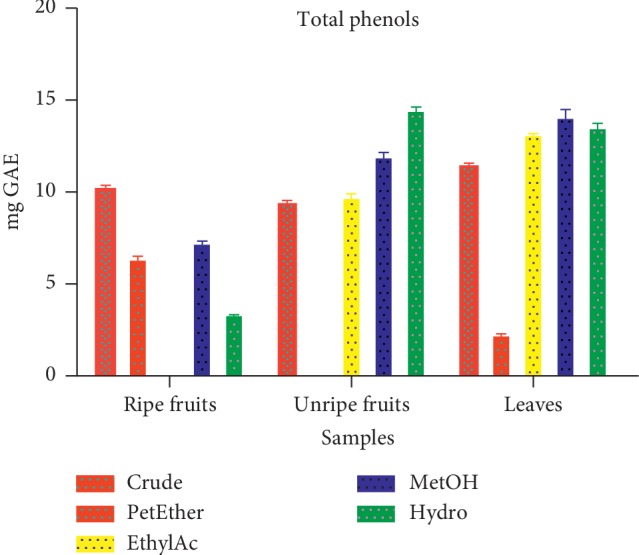
Total phenolic content of extract and fractions of *D. erecta*.

The total polyphenolic contents varied among the extracts and fractions. The mean highest total phenolic level was observed in the hydro fraction of unripe fruit with a corresponding value of 14.54 ± 0.29 mg GAE/100 g, whilst the lowest value of 2.20 ± 0.15 mg GAE/100 g was recorded in petroleum ether fraction of the leaves. This is presented in Figure 4.

#### 3.1.6. Total Flavonoid Content

**Figure 5:**
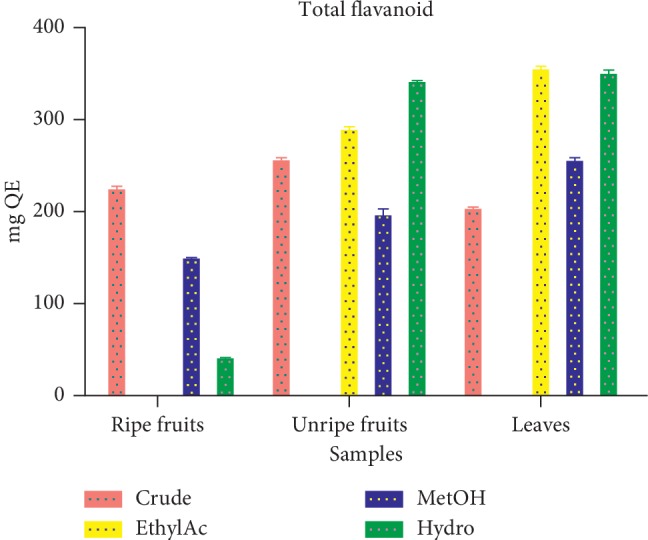
Total flavonoid content of extract and fractions of *D. erecta*.

The highest total flavonoid content was observed in ethyl acetate fraction of the leaves (343.49 ± 3.45 *μ*g QE/100 g). This is followed by hydro fraction of leaves (338.70 ± 4.18 *μ*g QE/100 g), hydro fraction of unripe fruit (330.08 ± 1.92 *μ*g QE/100 g), ethyl acetate fraction of unripe fruit (280.08 ± 3.45 *μ*g QE/100 g), crude extract of unripe seeds (248.60 ± 2.25 *μ*g QE/100 g), crude extract of ripe seeds (218.00 ± 3.45 *μ*g QE/100 g), methanolic fraction of ripe seeds (146.17 ± 0.96 *μ*g QE/100 g), and hydro fraction of unripe seeds (41.76 ± 0.96 *μ*g QE/100 g) (Figure 5).

#### 3.1.7. Total Tannins Content (TTC)

**Figure 6:**
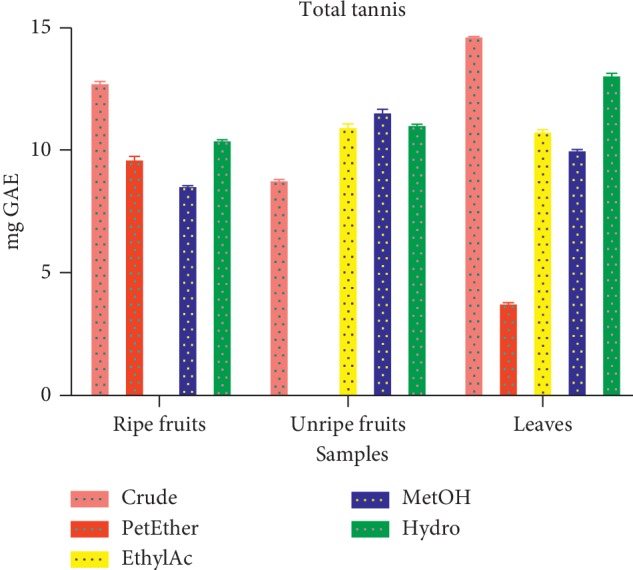
Total tannins content of extract and fractions of *D. erecta*.

The TTC was expressed in terms of gallic acid equivalent per 100 g of the extract. The TTC varied from 13.82 ± 0.04 mg GAE/100 g to 3.55 ± 0.07 mg GAE/100 g (Figure 6).

#### 3.1.8. DPPH Free Radical Scavenging Activity

**Figure 7:**
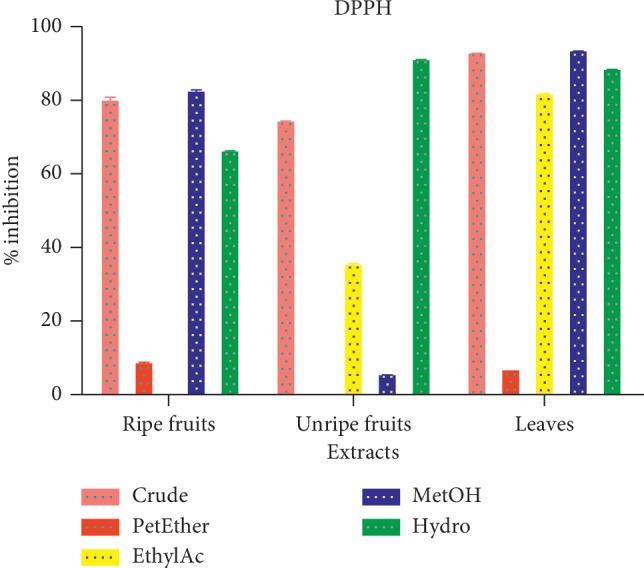
The DPPH free radical scavenging activity of *D. erecta*.

The DPPH free radical scavenging activity was expressed as percentage (%). The highest scavenging activity of 90.0% was registered for methanolic fraction of the leaves, whilst the lowest methanolic fraction of 5.4% was seen in unripe fruit (Figure 7).

#### 3.1.9. Antimicrobial Activity

The antimicrobial activity assay of the ripe fruit, unripe fruit, and leaves of *D. erecta* proved negative for all the crude, hydro, and methanol fractions with no recorded antimicrobial activity against the test organisms. The positive control (standard antibiotic), however, recorded positive responses to the test organisms.

### 3.2. Discussion

In the present study, we carried out phytochemical investigation on *Duranta erecta* with the view of determining its usefulness in traditional medicine. The leaves and ripened fruits contained tannins, glycosides, saponins, flavonoids, and triterpenoids, whilst the unripe fruits contained all the aforementioned in addition to alkaloids (Table 1). The absence of alkaloids in the leaves is not consistent with earlier reports [9, 18] that detected an appreciable level of alkaloids in *D. erecta* leaves. Phytochemicals play important role by combining with nutrients and dietary fibre to us against diseases though some have bitter taste. Alkaloids have been shown to have antioxidant properties via alleviating H2O2-induced oxidative damage [19]. Alkaloids have also been implicated in antimalarial activity of many plants by blocking protein synthesis in *Plasmodium falciparum* [20]. Tannins are known potent antioxidants, and this activity is achieved by chelation of metal ions such as Fe (II) and interfering with one of the reaction steps in the Fenton reaction thereby retarding oxidation [21]. Saponins possess antioxidant potential through hydrogen peroxide scavenging capability [21]. Flavonoids have the ability to inhibit lipid peroxidation by sugar substitutions in the phenolic C ring [19]. Triterpenoids and steroid saponins have been found detrimental to *P. falciparum* [19]. This goes to buttress the use of *D. erecta* in malaria in folk medicine. The presence of these phytochemicals account for their multipurpose in traditional medicine.

The concentration levels of Ni and Pb were below detectable limits of 1 × 10–5 mg·L^−1^ for the leaves and fruits. The highest mean concentration for Fe (2.17 ± 0.05 mg·L^−1^) was found in leaves (raw) and the lowest (0.33 ± 0.01 mg·L^−1^) in the ripened fruit extracts (Table 2). The permissible limit of Fe in medicinal plants is 20 mg·L^−1^ [22]. The concentration of Zn in the analysed samples ranged from 1.35 ± 0.04 mg·L^−1^ to 0.40 ± 0.02 mg·L^−1^. The highest concentration level of Zn was recorded in the raw leaf samples. These values were within FAO/WHO permissible limits of 27.4 mg·L^−1^ in medicinal plants and were regarded as safe. The concentration of Cu in the samples varied. The highest Cu level of 0.11 ± 0.01 mg·L^−1^ was observed in ripened fruit, followed by unripe 0.04 ± 0.01 mg·L^−1^ and leaves 0.02 ± 0.01 mg·L^−1^. The permissible limit of Cu in plants recommended by WHO is 10 mg·kg^−1^. In all the analysed samples, concentration of Cu recorded was below the permissible limit.

The use of medicinal plants for healthcare presents a way to humans to get exposed to trace elements and heavy metals. Major and trace elements have significant roles in combating a variety of human ailments and diseases [23]. Also, beyond certain limits, these may present serious health hazards to humans. The knowledge on minerals and heavy metal content is essential for safe use of medicinal plants. All the elements evaluated were within the WHO/FAO permissible limits. The presence of these elements, and in the appropriate amounts, in the plant confers useful values on this plant. Fe is an important trace element, and iron-protein mixtures play a vital role in metabolism. It is essential in the biosynthesis of haemoglobin and transport of oxygen and electrons throughout the body [24]. Zn is a component of many metalloenzymes, especially enzymes which play a central role in nucleic acid metabolism. It is also essential in bone formation, wound healing, brain development, and normal growth processes [25]. Cu is an essential component of many enzymes, and it plays a significant role in a wide range of physiological processes including Fe utilization, free radical elimination, bone and connective tissues development, and melanin production [24].

The presence of phytochemicals was confirmed by spectroscopic studies showing the characteristic spectra obtained in the ultraviolet and visible spectrum. The UV-Vis spectra of the extracts of the various parts had absorption maxima in the region of 203.0–385.1 nm, 202–423.0 nm, and 203.0–379.0 nm for the leaves, unripe fruits, and ripe fruits, respectively. These absorption bands are characteristic for flavonoid and its derivatives. The flavonoid spectra mainly consist of two absorption maxima in the ranges of 230–285 nm and 300–350 nm [26]. Similar results were confirmed in an earlier study [27] that reported UV-Vis spectrum of leaf extract in the absorption range of 270 and 340 nm.

The FTIR spectrum was used to establish identity of the functional groups present in extract based on the peak values in the region of infra-red radiation. The results of FTIR analysis revealed the presence of alcohols, phenols, alkanes, aldehydes, ketones, aromatics, aliphatic amines, aromatic amines, amides, carboxylic acids, esters, nitro compounds, alkynes, primary and secondary amines, and alkyl halides (Figures 1–3). These were confirmed by FTIR spectroscopic analysis that predicted the presence of the following groups: C-Br, O-H, C-H, C=C, C=O, C≡C, N-H, C-H, C-N, C=0. The presence of the various characteristic functional groups may be responsible medicinal properties of *D. erecta*.

The hydro fraction of the unripe fruit recorded the highest total phenolic content (14.54 ± 0.29 mg GAE/100 g), whilst the petroleum ether fraction of the leaves exhibited the lowest value (2.20 ± 0.15 mg GAE/100 g; Figure 4). The polyphenolic content in the fraction depends on the polarity of the extraction solvent used. High solubility of phenols in polar solvents provides high concentration of these compounds when polar solvent is used in the extraction process [28]. Polyphenolic compounds are important plant constituents due to their scavenging ability. The scavenging ability is by virtue of the hydroxyl groups which has high affinity for hydrogen atom, thereby conferring antioxidative property on the compounds. The mode of action of phenolic compounds in free radical moping activity is by inactivating lipid free radicals or by preventing the decomposition of hydroperoxides into free radicals [29]. The antioxidant property makes such compounds useful in the management of oxidative stress in humans.

From Figure 5, the total flavonoid content varied from 343.49 ± 3.45 *μ*g QE/100 g to 47 ± 0.96 *μ*g QE/100 g. The highest total flavonoid content was recorded in the ethyl acetate fraction of the leaves and the lowest in the hydro fraction of unripe fruits. The concentration of flavonoids in extract depends on the polarity of solvents and the part of plant material used for extractions [30]. The highest concentration of flavonoids was recorded in solvent of moderate polarity. The antioxidative properties of flavonoids can be linked to several different mechanisms, such as scavenging of free radicals, chelation of metal ions, such as iron and copper, and inhibition of enzymes responsible for free radical generation [29] Flavonoids also possess antibacterial activity. The antibacterial mechanisms of action of selected flavonoids are attributed to inhibition of DNA gyrase, cytoplasmic membrane function and complexing with bacterial cell wall, and licochalcones A and C energy metabolism [31].

The crude extract of the leaves recorded the highest concentration of tannins level 13.82 ± 0.04 mg GAE/100 g, while the least (3.55 ± 0.07 mg GAE/100 g) was observed in petroleum ether fraction of the leaves. Comparing our data with a similar work [22, 32] done elsewhere in Africa, the current data showed higher tannin levels. The differences could be partly due to climatic conditions and the part of the plant used in the extraction. Tannins inhibit lipid peroxidation by downregulating activity of cyclo-oxygenase enzyme through inflammation pathway. However, tannins interact with protein molecules forming large cross-linked complexes, thereby rendering them indigestible.

DPPH is a stable radical relative to other *in vitro*-generated free radicals such as hydroxyl radical and superoxide anion. DPPH has an added advantage of being not affected by certain side reactions, such as metal-ion chelation and enzyme inhibition. It is used to evaluate the free radical mopping capacity of compounds. In this study, the DPPH scavenging activity ranged from 90.0% to 5.35% with the leaf showing the highest activity. The high scavenging activity associated with the leaves explain their relevance in traditional medicine. Free radical scavenging is one of the known mechanisms by which antioxidants inhibit lipid peroxidation. This is associated with the activity of antioxidant enzymes such as superoxide dismutase, catalase, and glutathione peroxidase.

From this study (Tables 4–6), the extract/fractions did not show antimicrobial activity against the test organism. This is not consistent with the other previous studies that recorded antimicrobial activity of *Duranta erecta* against *Pseudomonas aeruginosa*, *Proteus mirabilis*, *Salmonella typhi*, *Escherichia coli*, and *Klebsiella pneumonia*. Phytochemicals such as flavonoids, triterpenoids, and alkaloids are widely known to exhibit some level of antimicrobial activity against some bacteria [33]. Biological activity of the extract is more effective at higher concentrations, and antimicrobial activity decreases considerably with reduction in the concentration of the extract [22].

## 4. Conclusion

The leaves and fruits of *Duranta erecta* contained important phytochemicals including flavonoids, phenols, saponins, sterols, tannins, alkaloids, and triterpenoids and minerals such as Fe, Zn, and Cu which were within FAO/WHO permissible limits. The various parts demonstrated important antioxidant and beneficial properties which are necessary for its usefulness in traditional medicine. The results of FTIR analysis confirmed the presence of alcohols, phenols, alkanes, aldehydes, ketones, aromatics, aliphatic amines, aromatic amines, amides, carboxylic acids, esters, nitro compounds, alkynes, primary and secondary amines, and alkyl halides.

## Figures and Tables

**Table 1:** Phytochemical constituents of *D. erecta*.

Test	Leaves	Unripe fruits	Ripe fruits
Tannins	+	+	+
Glycosides	+	+	+
Alkaloids	−	+	−
Coumarins	−	−	−
Saponins	+	+	+
Flavonoids	+	+	+
Triterpenoids	+	+	+
Sterols	+	+	+

*Note.* +, present; −, not detected.

**Table 2:** Heavy metal content of raw plant materials and extracts.

Material	Metals				
	Fe (mg·L^−1^)	Zn (mg·L^−1^)	Ni (mg·L^−1^)	Cu (mg·L^−1^)	Pb (mg·L^−1^)
Unripe fruit raw	1.97 ± 0.06	0.70 ± 0.01	BDL	0.04 ± 0.01	BDL
Unripe fruit extract	0.64 ± 0.04	0.40 ± 0.02	BDL	BDL	BDL
Ripe fruit, raw	2.03 ± 0.06	0.64 ± 0.01	BDL	0.11 ± 0.01	BDL
Ripe fruit, extract	0.33 ± 0.01	0.43 ± 0.01	BDL	BDL	BDL
Leaves, raw	2.17 ± 0.05	1.35 ± 0.04	BDL	0.02 ± 0.01	BDL
Leaves, extract	0.96 ± 0.03	1.26 ± 0.045	BDL	BDL	BDL

*Note*. BDL, below detection limit; detection limit, 0.00001 mg·L^−1^.

**Table 3:** UV-Vis peaks and absorption range.

Extract/fractions	Number of peaks	Wavelength range (nm)
Leaves		
Petroleum ether fraction	5	203.0–268.1
Ethyl acetate fraction	5	204.0–226.0
Methanol fraction	5	204.2–215.9
Hydro fraction	3	285.2–385.1
Crude extract	2	284.0–382.9
Unripe		
Petroleum ether fraction	5	205.9–218.1
Ethyl acetate fraction	5	204.0–226.0
Methanol fraction	3	203.7–322.3
Hydro fraction	5	287.1–381.5
Crude extract	5	286.2–4231
Ripe		
Petroleum ether fraction	5	203.0–217.3
Ethyl acetate fraction	5	207.0–223.0
Methanol fraction	5	201.5–384.1
Hydro fraction	4	286.9–376.9
Crude extract	3	285.9–379.0

**Table 4:** Antimicrobial activity of fractions of ripe fruits against test organisms.

Extract	TO 1	TO 2	TO 3	TO 4	TO 5	TO 6	TO 7	TO 8	TO 9
Ex 1	—	—	—	—	—	—	—	—	—
Ex 2	—	—	—	—	—	—	—	—	—
Ex 3	—	—	—	—	—	—	—	—	—
Ctrl N	—	—	—	—	—	—	—	—	—
Ctrl P	2.5 ± .15	2.0 ± .23	2.3 ± .17	1.0 ± .06	1.7 ± .33	2.6 ± .10	2.1 ± .15	1.9 ± .33	2.2 ± .41

*Note*. TO: test organism; Ex: extract fractions; Ctrl N negative control; Ctrl P positive control; TO 1: *Pseudomonas aeroginosa*; TO 2: *Proteus mirabilis*; TO 3: *Salmonella typhi*; TO 4: *Staphylococcus saprohyticus*; TO 5: *Candida albicans*; TO 6: *Staphylococcus aureus*; TO 7: *Escherichia coli*; TO 8: *Klebsiella pneumonia*; TO 9: *Paratyphi B*; Ex 1: crude extract; Ex 2: hydro fraction; Ex 3: methanol fraction.

**Table 5:** Antimicrobial activity of fractions of leaves against test organisms.

Extract	TO 1	TO 2	TO 3	TO 4	TO 5	TO 6	TO 7	TO 8	TO 9
Ex 1	—	—	—	—	—	—	—	—	—
Ex 2	—	—	—	—	—	—	—	—	—
Ex 3	—	—	—	—	—	—	—	—	—
Ctrl N	—	—	—	—	—	—	—	—	—
Ctrl P	2.5 ± .15	2.0 ± .23	2.3 ± .17	1.0 ± .06	1.7 ± .33	2.6 ± .10	2.1 ± .15	1.9 ± .33	2.2 ± .41

*Note*. TO: test organism; Ex: extract fractions; Ctrl N negative control; Ctrl P: positive control; TO 1: *Pseudomonas aeroginosa*; TO 2: *Proteus mirabilis*; TO 3: *Salmonella typhi*; TO 4: *Staphylococcus saprophyticus*; TO 5: *Candida albicans*; TO 6: *Staphylococcus aureus*; TO 7: *Escherichia coli*; TO 8: *Klebsiella* pneumonia; TO 9: *Paratyphi B*; Ex 1: crude extract; Ex 2: hydro fraction; Ex 3: methanol fraction.

**Table 6:** Antimicrobial activity of fractions of unripe fruits against test organisms.

Extract	TO 1	TO 2	TO 3	TO 4	TO 5	TO 6	TO 7	TO 8	TO 9
Ex 1	—	—	—	—	—	—	—	—	—
Ex 2	—	—	—	—	—	—	—	—	—
Ex 3	—	—	—	—	—	—	—	—	—
Ctrl N	—	—	—	—	—	—	—	—	—
Ctrl P	2.5 ± .15	2.0 ± .23	2.3 ± .17	1.0 ± .06	1.7 ± .33	2.6 ± .10	2.1 ± .15	1.9 ± .33	2.2 ± .41

*Note*. TO: test organism; Ex: extract fractions; Ctrl N: negative control; Ctrl P: positive control; TO 1: *Pseudomonas aeroginosa*; TO 2: *Proteus mirabilis*; TO 3: *Salmonella typhi*; TO 4: *Staphylococcus saprophyticus*; TO 5: *Candida albicans*; TO 6: *Staphylococcus aureus*; TO 7: *Escherichia coli*; TO 8: *Klebsiella pneumonia*; TO 9: *Paratyphi B*; Ex 1: crude extract; Ex 2: hydro fraction; Ex 3: methanol fraction.

## Data Availability

The data used to support the findings of this study are available from the corresponding author upon request.
